# Recurrent Pneumothorax Revealing Lymphangioleiomyomatosis in a Young Woman: A Case Report

**DOI:** 10.1002/ccr3.71618

**Published:** 2025-12-09

**Authors:** Qihang Zhang, Tianli Zuo, Yongjun He, Wei Zheng

**Affiliations:** ^1^ Santai People's Hospital, Affiliated Santai Hospital of North Sichuan Medical College Mian Yang P. R. China

**Keywords:** high‐resolution computed tomography, HMB‐45, Lymphangioleiomyomatosis, mTOR, pneumothorax, vascular endothelial growth factor‐D

## Abstract

Lymphangioleiomyomatosis (LAM) should be considered in young women presenting with recurrent pneumothorax. Early diagnosis through high‐resolution computed tomography(HRCT)and histopathological analysis is essential for timely management and improved patient outcomes.

**Abbreviations**
CD3: Cluster of Differentiation 3
CD34: Cluster of Differentiation 34
CT: Computed Tomography
HE: Hematoxylin and Eosin
HMB‐45: Human Melanoma Black‐45
HRCT: High‐Resolution Computed Tomography
LAM: Lymphangioleiomyomatosis
mTOR: Mammalian Target of Rapamycin
SMA: Smooth Muscle Actin
TSC1: Tuberous Sclerosis Complex 1
TSC2: Tuberous Sclerosis Complex 2

## 1 Introduction

Lymphangioleiomyomatosis (LAM) is a rare cystic lung disease mainly affecting women of reproductive age. Recent population‐based studies estimate the prevalence of LAM to be approximately 23.5 cases per million adult females [1]. It is characterized by abnormal proliferation of smooth muscle‐like cells, leading to diffuse pulmonary cysts and recurrent pneumothorax [2].

LAM can occur sporadically or in association with tuberous sclerosis complex (TSC), involving mutations in the *TSC1* or *TSC2* genes and dysregulation of the mTOR pathway [3]. High‐resolution computed tomography(HRCT) typically reveals multiple thin‐walled cysts throughout both lungs. Immunohistochemical staining is useful for diagnosis, with markers such as HMB‐45, SMA, and D2‐40 commonly positive [3, 4].

The diagnostic approach to LAM has been revolutionized by the discovery of vascular endothelial growth factor‐D (VEGF‐D). In a patient with compatible HRCT findings, a serum VEGF‐D level ≥ 800 pg/mL is considered diagnostic, often obviating the need for lung biopsy.

Recent advances have shown that mTOR inhibitors, particularly sirolimus, can significantly improve or stabilize lung function, reduce the frequency of pneumothorax and chylous effusions, and delay disease progression in patients with LAM [5, 6]. For end‐stage LAM complicated by respiratory failure, lung transplantation remains a definitive option. While recurrence of LAM post‐transplant is rare, cases have been documented. One report described recurrent LAM in the transplanted allograft of a woman receiving a single lung transplant [7]. Another case from Pittsburgh reported symptomatic allograft recurrence several years post‐transplant in a LAM patient [8].

LAM should be considered in young women presenting with recurrent pneumothorax, as early diagnosis and intervention can improve prognosis [9]. This report presents a case in which recurrent pneumothorax led to the diagnosis of LAM, highlighting the importance of combined imaging and histopathology in diagnosis.

## 2 Case Report

### 2.1 Case History / Examination

A 27‐year‐old non‐smoking woman was admitted with a 20‐day history of chest tightness and shortness of breath. Her symptoms had initially started 20 days prior, when she was evaluated at a local hospital for right chest tightness and dyspnea. Chest computed tomography (CT) at that time revealed a right pneumothorax with multiple thin‐walled cysts, and she underwent right lung bullectomy with symptomatic improvement. She was discharged but developed recurrent symptoms 10 days later, which were initially overlooked. Three days before the current admission, her symptoms worsened, prompting referral to our department.

**FIGURE 1:**
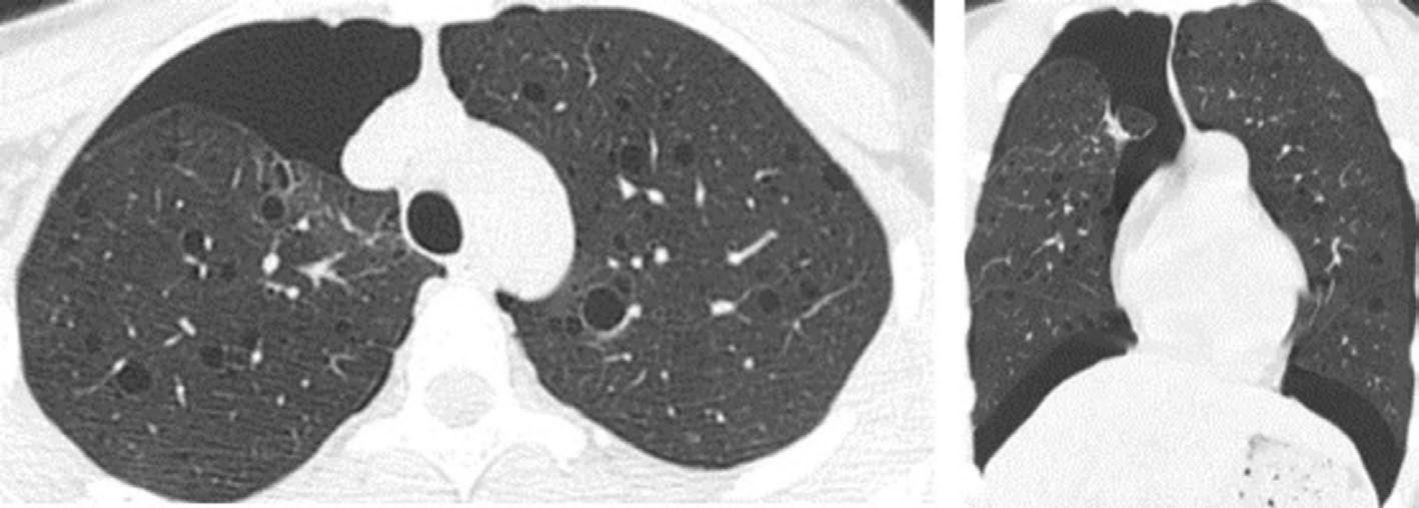
Chest CT scan obtained on April 25 demonstrates multiple cystic lesions in both lungs, bilateral fibrotic foci, and small pneumothoraces on both sides, with approximately 30% compression of the right lung and 20% compression of the left lung.

On admission (April 25, 2021), chest CT revealed bilateral pneumothoraces with approximately 30% compression of the right lung and 20% of the left lung, as well as multiple thin‐walled cysts in both lungs and subcutaneous emphysema of the right anterior chest wall (Figure 1).

Her past history included closed thoracic drainage for right pneumothorax 2 years prior. Laboratory studies were unremarkable.

On physical examination, there was decreased respiratory movement and tactile fremitus bilaterally, hyperresonant percussion, unclear lung lower borders and mobility, and palpable crepitus on the right anterior chest wall.

### 2.2 Differential Diagnosis, Investigations, and Treatment

Given the patient's age and clinical presentation, differential diagnoses included primary spontaneous pneumothorax, pulmonary Langerhans cell histiocytosis, Birt–Hogg–Dubé syndrome, and LAM. The population affected by lung Langerhans histiocytosis (PLCH) comprised adults aged 20–40, almost all of whom are heavy smokers. The cause of its onset is the accumulation of an abnormal type of immune cells (Langerhans cells) in the lungs. The main symptoms are dry cough, shortness of breath, and sometimes weight loss or fever. CT scans show nodules and irregularly shaped cysts, mainly in the upper lungs. Special laboratory tests on lung fluid or lung tissue can confirm the diagnosis. Birt–Hogg–Dubé syndrome (BHD) usually occurs in adults with a family history of lung or kidney diseases. The cause of the disease is a gene mutation (FLCN gene), which can lead to lung cysts, skin lumps, and sometimes kidney tumors. The main symptoms are recurrent lung collapse. Skin lesions (small lumps around the hair follicles) and renal tumors may also occur. CT scans show many thin‐walled cysts near the lung surface. Genetic testing can confirm FLCN mutations. Lymphangioleiomyomatosis (LAM) is more common in women of childbearing age. The main cause is the uncontrolled growth of abnormal muscle‐like cells in the lungs, resulting in air‐filled cysts. The main symptoms are shortness of breath, which worsens over time, repeated lung collapse, and sometimes there is milky white fluid around the lungs (chylothorax). CT scans show that many circular thin‐walled cysts are evenly distributed in both lungs. The detection of VEGF‐D in the blood can be helpful, and lung biopsy also confirmed this.

The patient received continuous nasal oxygen at 5 L/min, budesonide aerosol inhalation, and supportive care for water and electrolyte balance.

**FIGURE 2:**
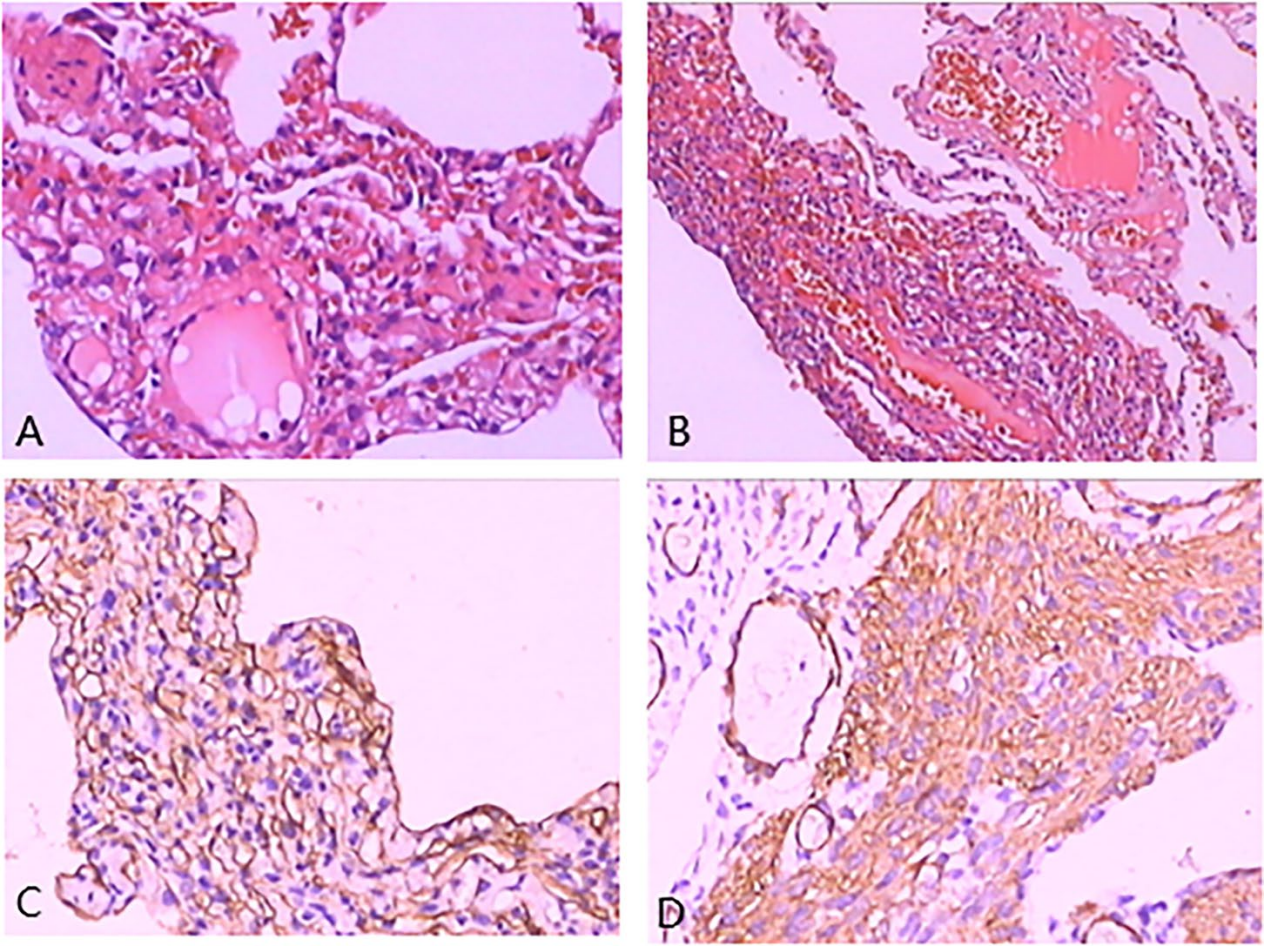
(A–B) Hematoxylin and eosin (H&E) staining reveals spindle‐shaped tumor cells with eosinophilic cytoplasm, lacking nuclear atypia or mitotic activity. The proliferating cells are epithelioid and arranged in bundles or networks. (C–D) Immunohistochemical staining shows positive reactivity for SMA, Desmin, Vimentin, HMB‐45, D2‐40, h‐caldesmon, CD34, and CD3, findings consistent with lymphangioleiomyomatosis (H&E, ×100).

On hospital day 3, a pathological review of external surgical specimens revealed cystic changes in the lung parenchyma, thinning of alveolar walls and septa, lymph node hyperplasia with lymphatic dilation and cystic expansion, and smooth muscle proliferation in lymph node walls (Figure 2).

Immunohistochemical staining was positive for SMA, Desmin, Vimentin, HMB‐45, D2‐40, h‐caldesmon, CD34, and CD3, findings consistent with LAM. Genetic testing for TSC1/TSC2 mutations was recommended to confirm the diagnosis. Serum VEGF‐D testing, TSC1/TSC2 genetic analysis, and abdominal imaging to evaluate for renal angiomyolipomas were recommended as part of the diagnostic workup. However, these investigations were **not completed**, as they were not locally available at the time of hospitalization and the patient declined transfer for further testing. The diagnosis of LAM in this case was therefore established on the basis of characteristic HRCT findings and confirmatory histopathological features obtained during surgery for pneumothorax.

## 3 Conclusion and Results (Outcome and Follow‐Up)

**FIGURE 3:**
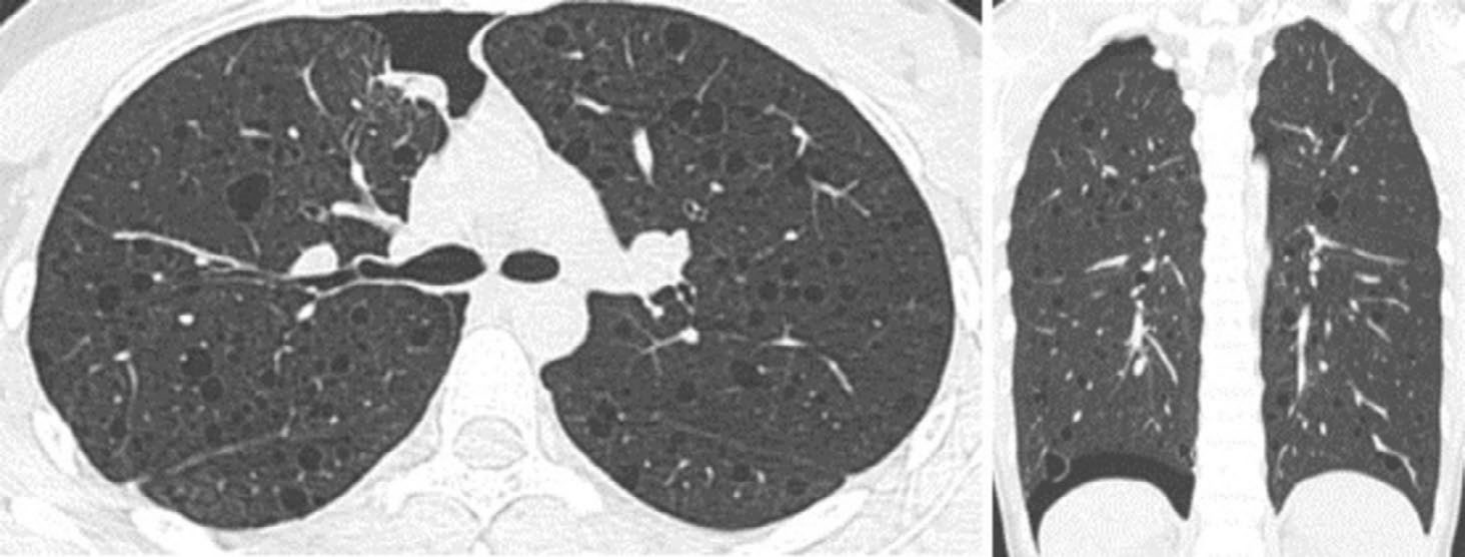
Chest CT scan performed on May 2 shows a significant reduction in the right pneumothorax, with residual lung compression of approximately 5%. There is complete resolution of the left pneumothorax and marked improvement in right anterior chest wall subcutaneous emphysema.

The patient's clinical status improved gradually with conservative management, including supportive care and close monitoring of respiratory function. The patient's respiratory rate was maintained between 18 and 20 beats per minute, blood oxygen saturation was kept above 95%, heart rate and blood pressure were both within the normal range, and breathing difficulties were relieved at the same time. A follow‐up chest CT performed on May 2, 2021, demonstrated a marked reduction in the right pneumothorax, with the right lung re‐expanding and the residual compression decreasing to approximately 5% of the lung volume. There was also complete resolution of the left pneumothorax, and a significant improvement in the right anterior chest wall subcutaneous emphysema, as shown in Figure 3. Based on these favorable imaging findings and clinical stability, the right chest drainage tube was removed. After an additional 24 h of careful observation for any signs of respiratory distress or recurrence, the patient was discharged from the hospital in stable condition.

At her most recent outpatient follow‐up visit, the patient remained asymptomatic, and her pulmonary function tests were within normal limits. She was advised to undergo ongoing clinical and radiological surveillance to monitor for any signs of recurrence or further progression of the disease, in accordance with current best practice for LAM management. Sirolimus therapy was not initiated because her lung function remained within the normal range and no progressive decline was observed, in line with current guideline recommendations for LAM management.

## 4 Discussion

Lymphangioleiomyomatosis (LAM) is a rare, diffuse cystic lung disease. Recent population‐based studies from European national referral centers estimate the prevalence to be approximately 23.5 cases per million adult females [1], suggesting that LAM is more common than previously recognized. The pathogenesis remains unclear, but studies suggest a relationship with estrogen levels in women [10], while cases in premenarcheal and postmenopausal women are rarely reported. Early‐stage manifestations include recurrent pneumothorax, dyspnea, and chylothorax; a minority of patients present with hemoptysis. Disease progression eventually leads to severe dyspnea, hypoxemia, and respiratory failure. Clinical manifestations are nonspecific and can be easily confused with spontaneous pneumothorax or pulmonary interstitial fibrosis in early stages, leading to misdiagnosis. Early HRCT plays an important role in diagnosis [11], revealing the extent and number of pulmonary cystic lesions to indicate disease progression, while definitive diagnosis depends on pathological examination. In most contemporary practice, serum VEGF‐D measurement is recommended as the initial non‐invasive diagnostic test, with levels ≥ 800 pg/mL considered virtually diagnostic of LAM [1]. In this case, VEGF‐D testing and TSC1/TSC2 genetic analysis were not performed because these investigations were not available locally, and the patient declined further referral. Renal imaging to assess for angiomyolipomas—another important diagnostic clue—was also not completed. Consequently, the diagnosis relied on the characteristic radiological features and the histopathological findings obtained during surgical management of pneumothorax. Mutations in *TSC1* and *TSC2* genes located on chromosomes 9 and 16 have been identified in patients with lymphangioleiomyomatosis. As tumor suppressor genes, mutation and inactivation of *TSC1*/*TSC2* enable cells to evade immune surveillance, promote tumor‐related protein expression, and contribute to tumorigenesis [12]. Currently, there is no effective curative treatment for LAM. Conventional treatment focuses on symptomatic relief, including long‐term oxygen therapy, hormonal inhalation, and thoracic drainage; however, these do not slow disease progression. Given estrogen's influence on LAM, anti‐estrogen therapies have been explored clinically. However, a clinical study of 36 LAM patients by Schiavina et al. [13] reported inconclusive results for anti‐estrogen therapy, with some patients experiencing disease progression. Recent studies have demonstrated that mTOR inhibitors, such as Sirolimus and Everolimus, can stabilize lung function and improve quality of life in LAM patients [14]. Lung transplantation remains the only effective treatment for advanced LAM; however, high cost and potential for recurrence limit its application, with few cases reported in China.

## Author Contributions

**Qihang Zhang:** visualization, writing – original draft, writing – review and editing. **Tianli Zuo:** conceptualization, data curation, formal analysis. **Yongjun He:** formal analysis, investigation, methodology. **Wei Zheng:** project administration, writing – original draft, writing – review and editing.

## Funding

The authors received no specific funding for this work.

## Ethics Statement

The authors have nothing to report.

## Consent

Written informed consent was obtained from the patient for publication of identifying images or other personal or clinical details of this case report.

## Conflicts of Interest

The authors declare no conflicts of interest.

## Data Availability

Data available on request due to privacy/ethical restrictions.
